# Hereditary Insanity

**Published:** 1852-03

**Authors:** 


### PATHOLOGY AND PRACTICE OF MEDICINE.

*Hereditary Insanity.*—DR. MOREAU (de Tours), Physician to the Bicêtre Hospital at Paris (for old insane men), has read before the Academy of Science, an important paper with the following title :—"On hereditary predisposition to cerebral affections.—Can this predisposition be recognized by any particular signs?" The author rests his reasoning principally on comparative anatomy; his views may be condensed as follows: Constant and invariable laws regulate the manner in which the organization of the parents is transmitted to the offspring; hence arises likeness. The latter is not handed down in the shape of a few isolated features, but by the transmission of two great series of organs, which series are perfectly defined and distinct. One of these series includes the external form and configuration; the other regulates nervous functions. The transmission takes place according to fixed laws: when one of the parents communicate one series, the second parent transmits the other.

Passing afterwards from animals to man, and applying the above laws to human beings in their pathological order, Dr. Moreau has found that, in the majority of cases, when an hereditary similarity to *one* of the parents has been made manifest by certain pathological alterations of that portion of the nervous system which ministers to the intellectual faculties, the distinctive characters of that series of organs which preside over the oxpression of the face—viz., the vulgar acceptation of likeness—will not fail to have been transmitted by the *other* parent. This assertion is supported by 164 cases in a given number of 192.

The author, therefore, considers as demonstrated: 1st. That the law of hereditary transmission, according to series of organs, is founded upon truth, within certain limits, both as regards men and animals. 2d. That the transmission of cerebral disturbance and of the physical likeness may be effected by either parent, but most often by one of them only. 3d. That a family being given, among whose stock there have been one or more individuals affected with insanity, it is very probable that the hereditary disease will settle in preference upon such of the children as have little or no physical likeness with the relatives in whom the disease has originated; and that the mental affection will, on the contrary, not affect that portion of the offspring who bear to those relatives a more or less striking physical likeness.

We would here merely remark, that the above assertions are well worthy of investigation and discussion, as it is evident that prophylaxis would in some degree become easy, if these positions turn out perfectly correct.—*London Lancet.*

